# Transcriptome Profiling during Sequential Stages of Cryopreservation in Banana (*Musa* AAA cv Borjahaji) Shoot Meristem

**DOI:** 10.3390/plants12051165

**Published:** 2023-03-03

**Authors:** Chaw Su Su Htwe, Subramani Rajkumar, Pooja Pathania, Anuradha Agrawal

**Affiliations:** 1 ICAR-National Bureau of Plant Genetic Resources, Pusa Campus, New Delhi 110012, India; 2 Division of Plant Genetic Resources, ICAR-Indian Agricultural Research Institute, Pusa Campus, New Delhi 110012, India

**Keywords:** cryobionomics, cryoprotective agent, gene expression, *Musa*, osmotic dehydration, RNA seq, transcriptome

## Abstract

Cryopreservation approaches have been implemented in gene banks as a strategy to back up plant genetic resource collections that are vegetatively propagated. Different strategies have been employed to effectively cryopreserve plant tissue. There is little information on the cellular processes and molecular adjustments that confer resilience to the multiple stresses imposed during a cryoprotocol. In the present work, the cryobionomics of banana (*Musa* sp.), a non-model species, was investigated through the transcriptomic approach using RNA-Seq. Proliferating meristems of in vitro explants (*Musa* AAA cv ‘Borjahaji’) were cryopreserved using the droplet-vitrification technique. Transcriptome profiling analysis of eight cDNA libraries including the bio-replicates for T0 (stock cultures (control tissue), T1 (high sucrose pre-cultured), T2 (vitrification solution-treated) and T3 (liquid nitrogen-treated) meristem tissues was carried out. The raw reads obtained were mapped with a *Musa acuminata* reference genome sequence. A total of 70 differentially expressed genes (DEGs) comprising 34 upregulated and 36 downregulated were identified in all three phases as compared to control (T0). Among the significant DEGs (>log FC 2.0), during sequential steps, 79 in T1, 3 in T2 and the 4 in T3 were upregulated and 122 in T1, 5 in T2 and 9 in T3 were downregulated. Gene ontology (GO) enrichment analysis showed that these significant DEGs were involved in the upregulation of biological process (BP-170), cellular component (CC-10) and molecular function (MF-94) and downregulation of biological process (BP-61), cellular component (CC-3) and molecular function (MF-56). The Kyoto Encyclopedia of Genes and Genomes (KEGG) pathway analysis showed that DEGs were involved in the biosynthesis of secondary metabolites, glycolysis/gluconeogenesis, MAPK signaling, EIN 3-lke 1 protein, 3-ketoacy-CoA synthase 6-like, and fatty acid elongation during cryopreservation. For the first time, a comprehensive transcript profiling during four stages of cryopreservation in banana were carried out, which will pave the way for devising an effective cryopreservation protocol.

## 1. Introduction

Cryopreservation is an ex situ conservation strategy, where tissues or explants of plant/animal germplasm are stored at ultra-low temperatures (−180 to −196 °C), usually using liquid nitrogen (LN). It is the most valuable method for the long-term conservation of genetic resources of vegetatively propagated, non-orthodox seeded, short-lived orthodox seeded species, threatened species of plants and biotechnologically important cell lines [1]. At these temperatures, nearly all physico-chemical reactions and time-related metabolic changes are arrested [2,3]. Plant tissues in the form of seeds, zygotic or somatic embryos, shoot meristems, dormant buds, pollen and cell cultures have been successfully regrown with high recovery rates, up to 2–3 decades [4,5]. While a plethora of cryoprotocols are published in the literature, very few of them have actually translated into the cryobanking of germplasm [5]. In addition to other factors, a major challenge for large-scale application of the cryotechnology for genetic resource conservation has been the need to overcome species- and genotype-specific responses, as most published protocols are based on a limited number of species and genotypes [6]. The new-age vitrification-based protocols are the most widely used for the cryopreservation of crop germplasm of vegetatively propagated species, in which major steps include preculture, osmoprotection, dehydration with a vitrification solution (cryoprotective agent, CPA), rapid cooling, rapid warming, dilution of the vitrification solution, and culturing the thawed tissues for regrowth [5]. These processes during a cryopreservation technique cause intrinsic abiotic stresses such as cytotoxicity of the high concentrations of CPAs, osmotic and dehydration stress, uncontrolled formation and growth of ice during freezing and thawing, generation of reactive oxygen species (ROS), etc. [7]. These stresses have been shown to cause damage to cryopreserved plant tissues such as vacuolization, abnormalities and ruptures of the cell nuclear membranes, cell lysis, and autophagic decomposition [8].

Cryobiomics is a recently emerging field wherein advances in biomolecular or ‘omics’ technology is being applied to investigate and interpret causal factors related to cryoinjury with loss of viability and/or risks of genetic instability [2]. Cryobionomics has been defined as an interdisciplinary subject encompassing information on the phenotype, histology, cytology, biochemistry and molecular biology of an organism to evaluate possible damages at cellular, biochemical or metabolic levels (cryoinjury), and studying the temporal shifts in gene expression that cause the disruption of normal regulatory mechanisms, growth and developmental sequences [2]. Cryobiomics research has the potential to shed light on the correlation between cryoinjury, viability and genetic stability by investigating changes in the genome, transcriptome, proteome and metabolome. This in turn would facilitate developing more generic cryoprotocols applicable to a wide range of species/genotypes.

Prerequisites to investigate cryobiomic changes at the molecular level are a robust and repeatable cryopreservation protocol for a given species/a genotype, and genome sequence information to compare the gene expression patterns. The present investigation was undertaken in banana (*Musa* sp.), which is a non-model crop plant where a large number of cryoprotocols exist such as the slow-freezing method in embryogenic suspension cultures, simple freezing of sucrose pre-cultured proliferating meristematic clumps, the vitrification method in vegetatively propagated plant species for proliferating meristems clumps/scalps and apical meristems [9], droplet vitrification of banana apical and proliferating meristems, and air-desiccation methods for seeds/zygotic embryos of diploid *Musa* spp.; see ref. [10,11,12]. Another important feature in banana is the existence of a large number of wild species, cultivars and genetic diversity in different genomic and ploidy groups due to which there is variability in response after cryopreservation, and some accessions still remain recalcitrant in terms of their post-thaw regrowth [10,13]. The response of cells after dehydration by high sucrose, CPA treatment, and freezing stress during LN exposure is very hard to differentiate through phenotypic expression. Except for the model plant *Arabidopsis*, no studies are available on the molecular response of cryopreserved shoot tip explants through transcriptome analysis [14,15,16].

Hence, the present study was conducted to understand the molecular mechanism during different steps of droplet-vitrification cryopreservation protocol in banana using one cultivar with known high post-thaw response [17]. Transcriptomes at stages of in vitro proliferation of shoot meristems, pre-culture in high sucrose, after CPA treatment and after LN freezing were identified using the RNA-seq method, and the data generated were analyzed to classify genes which were upregulated or downregulated with respect to their ontologies.

## 2. Results

### 2.1. Post-Thaw Regrowth after Cryopreservation

Among the different techniques used in the cryopreservation of proliferating shoot meristem, the droplet-vitrification technique found to be better performing. The mean shoot regrowth in banana cv. Borjahaji after 60 min Plant Vitrification Solution 2 (PVS2) was 95.0%, while in Plant Vitrification Solution 2 (PVS2) + Liquid Nitrogen (LN)-treated samples, it was 66.7% (17). Along with the control (T0), the initial stage of High Sucrose treatment (T1), PVS2 treatment (T2), and PVS2+LN treatment (T3) tissues were taken in duplicates for transcriptome profiling.

### 2.2. High-Throughput Sequencing and Alignment to Reference Genome

**Figure 1:**
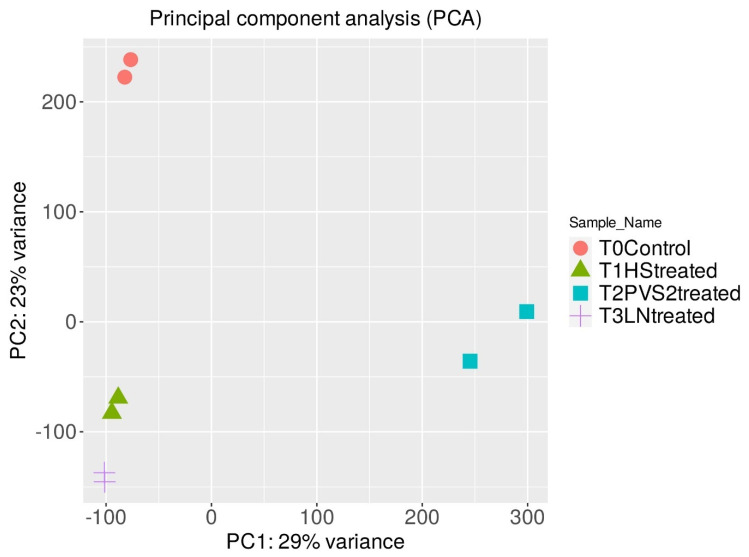
Principle component analysis (PCA) plot for RNA seq data of biological replicates of different stages of cryopreservation T0 (stock cultures (control), T1 (high sucrose pre-cultured), T2 (vitrification solution treated) and T3 (liquid nitrogen treated).

In the eight cDNA libraries after trimming by Cutadapt, 32.6 to 44.9 million clean reads were obtained (Table 1), with an average read length of 150 bp in bio-replicates. The quality scores (Q-score) of the trimmed data for all samples were above 30 (data not shown). Cleaned reads mapped to the banana reference genome resulted in alignment rates ranging from 72% to 93%, with the GC content of all samples between 48 and 53% (Table 1). The FPKM (fragments per kilobase of transcript per million reads) values of the predicted transcript were used to construct PCA plots to test the consistency of biological replicates. The PCA plot (Figure 1) showed the expected clustering of replicates of each stage with insignificant variation within the bio-replicates. The larger variation between the stages of cryopreservation clearly indicate that the pattern of variation is due to treatments during different cryopreservation stages.

#### Quantification of Differently Expressed Genes (DEGs)

**Figure 2:**
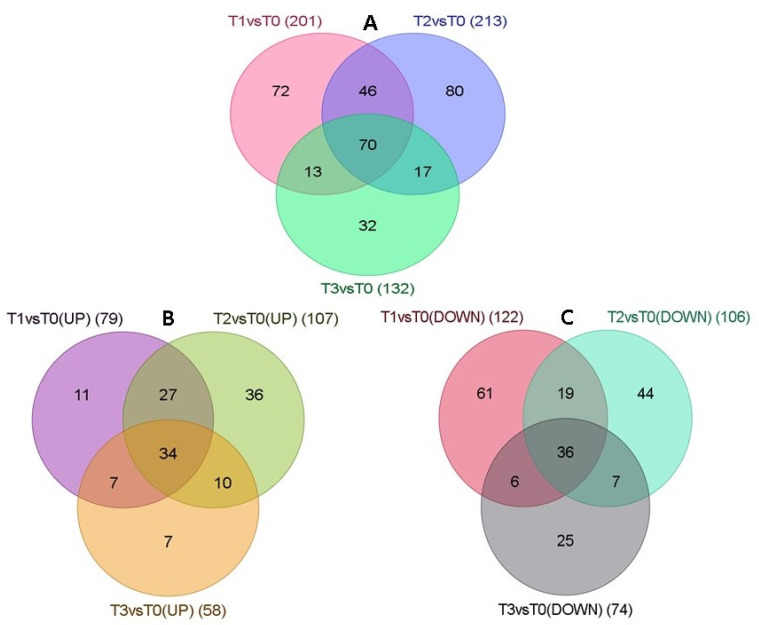
Venn diagrams of (**A**) Total number of DEGs, (**B**) Upregulated DEGs, and (**C**) Downregulated DEGs, T0—Control, T1—High sucrose treatment, T2—PVS2 treatment, T3—PVS2+LN treatment.

Multiple and uniquely mapped clean reads were used for quantification of the transcript. Table 2 shows the significantly important DEGs (Padj < 0.05) obtained based on analysis using the DESeq2 package. As compared to the control tissue (T0), 79, 107 and 58 upregulated and 122, 106 and 74 downregulated DEGs were recorded in tissues from T1, T2 and T3, respectively (Table 2, Supplementary Table S1). In contrast to control tissue versus each sequential stage during cryopreservation, the changes in DEGs within the three stages were significantly lower (8 in T2 vs. T1, 13 in T3 vs. T2, 17 in T3 vs. T1) (Table 2). Figure 2 provides the co-expression analysis of DEGs for all three stages (T1, T2 and T3) of cryopreservation as compared to control, depicting the total expressed DEGs (Figure 2A), upregulated DEGs (Figure 2B) and downregulated DEGs (Figure 2C). Compared to control tissue (T0), all the treatments (T1, T2, T3) have 70 common DEGs, out of which 34 were upregulated and 36 were downregulated (Figure 2B,C, Supplementary Table S1).

**Figure 3:**
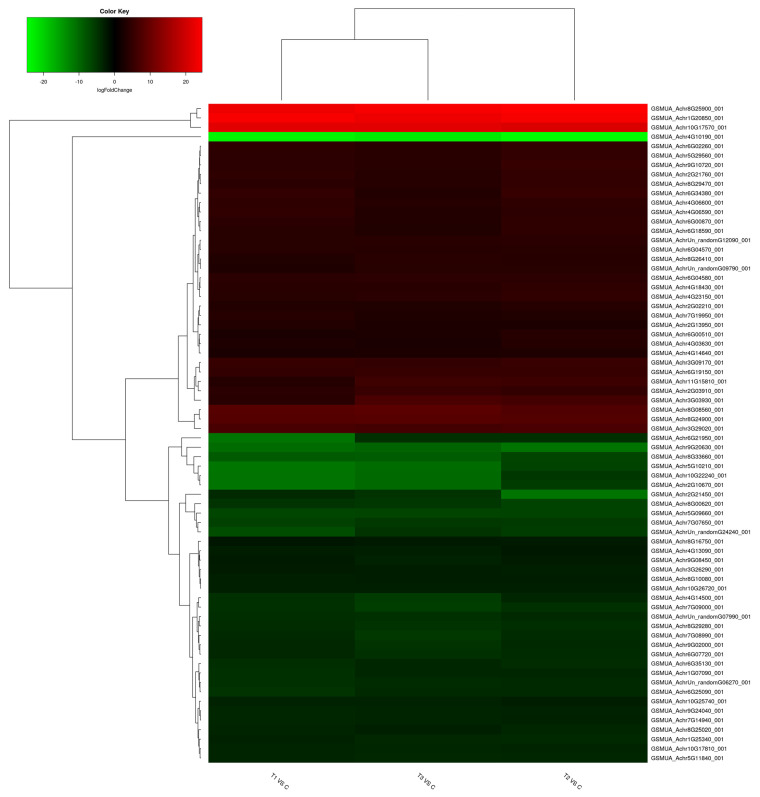
The heatmap profiling of common DEGs in three stages of cryopreservation: T1 vs. T0 (high sucrose treatment), T2 vs. T0 (PVS2 treatment) and T3 vs. T0 (PVS2+LN treatment). The different colors showed the values of log FC values for each DEG.

**Figure 4:**
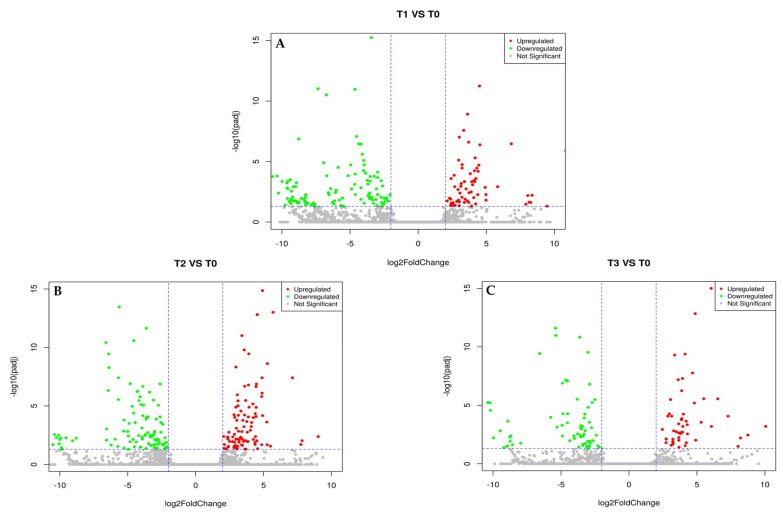
Volcano plots comparing all upregulated and downregulated DEGs in (**A**) T1 vs. T0 (high sucrose treatment), (**B**) T2 vs. T0 (PVS2 treatment), and (**C**) T3 vs. T0 (PVS2+LN treatment). Red points indicate upregulated DEGs (log FC > 2). Green points show downregulated DEGs (log FC < −2). Gray points represent non-significant DEGs.

The heatmap of 70 common DEGs (Figure 3) provides a visual depiction of the gradient and intensity of upregulated and downregulated genes in response to the sequential steps during cryopreservation. Figure 4 shows the volcano plots for all the DEGs, upregulated (log FC > 2.0) and downregulated (log FC < −2.0) based on their cut-off value. 

### 2.3. Functional Annotation and Enrichment of Common DEGs

The GO analysis of commonly found DEGs in all three treatments in comparison to control tissue that were significantly upregulated consisted of transferase activity, transcription factor and an uncharacterized protein, while oxidoreductase activity was significantly downregulated (Supplementary Table S2). Glycosyltransferase family 61 protein with a 20-fold increase in the transcript level was the highest among the upregulated DEGs. Some DEGs showed a variation in fold expression with different treatments. Among the upregulated common DEGs, the gene for calcium ion binding protein increased two-fold from T1 to T3. Similarly, among the downregulated common DEGs, fatty acid biosynthesis gene decreased three-fold and GRAM domain-containing protein decreased two-fold from T1 to T2.

#### 2.3.1. Functional Annotation and Enrichment of DEGs in High Sucrose Treatment

**Figure 5:**
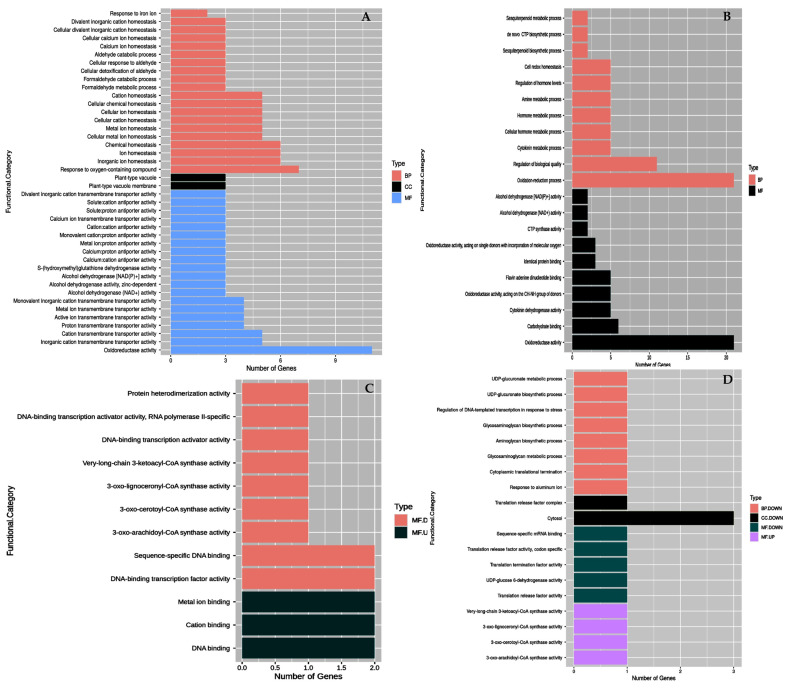
Histogram of Gene Ontology (GO) classification of significant DEGs (**A**) T1 vs. T0 (upregulated), (**B**) T1 vs. T0 (downregulated), (**C**) T2 vs. T1 (up and downregulated), (**D**) T3 vs. T2 (up and downregulated). (BP = Biological processes, MF = Molecular functions, CC = Cellular components).

In T 1(high-sucrose treatment) vs. T0, upregulated DEGs related to (i) transcription factors: NAC, basic leucine zipper Domain (bZIP); (ii) stress responsive proteins: ETHYLENE INSENSITIVE 3-like 1 protein (EIN3-like), F-box domain containing protein, late embryogenesis abundant protein (LEA5-D), APETALA2/ETHYLENE RESPONSIVE FACTOR (AP2/ERF) domain-containing proteins, small heat shock protein (SHSP-like); (iii) vacuolar cation/proton exchangers and very-long-chain 3-oxoacyl-CoA synthase (fatty acid biosynthesis process); (iv) enzymes: alcohol dehydrogenases (ADHs); and (v) signal transduction: protein kinases and protein phosphatase, which are involved in osmotic and hypoxia stress response (Supplementary Table S3). The most enriched category-wise GO terms among the highest 20 terms were ‘response to oxygen-containing compound’, ‘homeostasis’, ‘formaldehyde metabolic and catabolic process’, ‘response to aldehyde’, etc. in the biological process (BP), ‘oxidoreductase activity’, ‘transmembrane transporter activity’, ‘alcohol and glutathione dehydrogenase activity’, ‘antiporter activity’, etc. in the molecular function (MF) and ‘plant type vacuole’ and ‘plant type vacuole membrane’ in the cellular component (CC) (Figure 5A). Among the downregulated DEGs, enriched GO terms were ‘oxidation reduction process’, ‘regulation of biological quality’, ‘metabolic process’, etc. in BP and ‘oxidoreductase activity’, ‘carbohydrate binding’, ‘cytokinin and alcohol dehydrogenase activities’ etc. in MF (Figure 5B).

#### 2.3.2. Functional Annotation and Enrichment of DEGs in PVS2 Treatment

In T2 vs. T1, proteins namely putative polyol transporter 1, C3H1-type domain-containing protein and zinc finger A20 and AN1 domain-containing stress-associated protein 8-like were upregulated, while nuclear transcription factor Y subunit C-6-like, and very-long-chain 3-oxoacyl-CoA synthase activities were downregulated (Supplementary Table S3). The GO enrichment analysis among different treatments revealed that T2 vs. T1 led to an upregulation of ‘DNA, cation and metal ion binding’ activities in MF (and downregulation of ‘DNA-binding transcription activator activity’, ‘sequence-specific DNA binding’, ‘3-oxo-arachidoyl-, cerotoyl-, lignoceronyl-CoA synthase activities’, ‘DNA-binding transcription activator activities’ in MF (Figure 5C).

#### 2.3.3. Functional Annotation and Enrichment of DEGs in PVS2+LN Treatment

In T3 vs. T2, there was an upregulation of nuclear transcription factor Y subunit C-6-like, nucleolar GTP-binding protein and very-long-chain 3-oxoacyl-CoA synthase activities, whereas downregulation occurred in putative polyol transporter, UDP-glucose 6-dehydrogenase, EIN 3-like 1 protein and eRF1_3 domain-containing protein like activities (Supplementary Table S3). Upregulated DEGs were mostly associated with 3-oxo-arachidoyl-CoA synthase, 3-oxo-cerotoyl-CoA synthase, 3-oxo-lignoceronyl-CoA synthase and very-long-chain 3-ketoacyl-CoA synthase activities in MF, and downregulated DEGs were induced in ‘response to aluminum ion’, ‘UDP-glucuronate metabolic’, ‘biosynthetic process’, and ‘glycosaminoglycan metabolic and biosynthetic process’ in BP, ‘cytosol’ and ‘translation release factor activity’ in CC and ‘translation release factor activity’, ‘sequence-specific mRNA binding’ and ‘UDP-glucose 6-dehydrogenase activity’ in MF (Figure 5D).

### 2.4. KEGG Pathway Analysis

**Figure 6:**
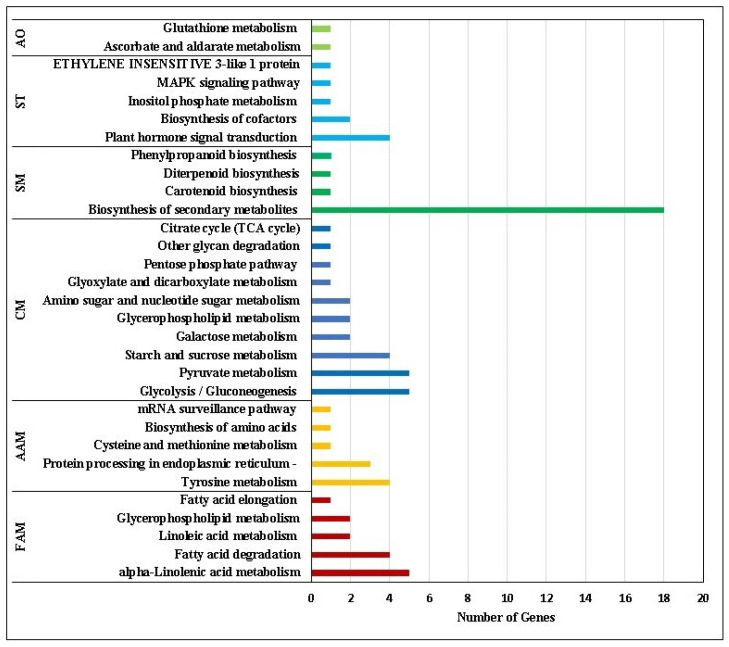
Histogram of KEGG function classification of DEGs T1 vs. T0 FAM—Fatty Acid Metabolism, AAM—Amino Acid Metabolism, CM—Cellular Mechanisms, SM—Secondary Metabolites, ST—Signal Transduction, AO—Antioxidant Activity.

**Figure 7:**
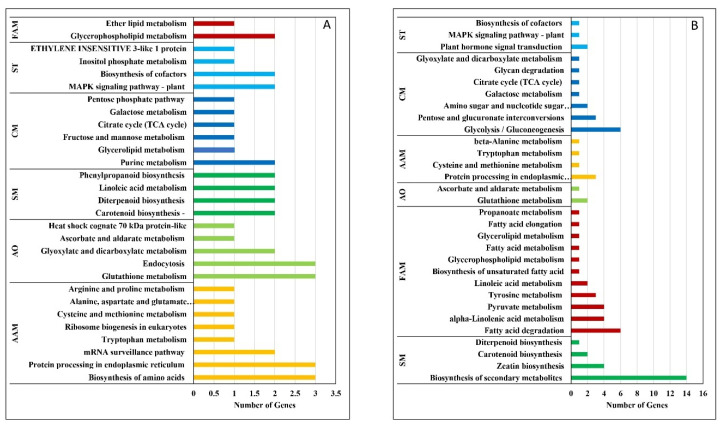
Histogram of KEGG function classification of DEGs (**A**). T2 vs. T0 (**B**). T3 vs. T0. FAM—Fatty Acid Metabolism, AAM—Amino Acid Metabolism, CM—Cellular Mechanisms, SM—Secondary Metabolites, ST—Signal Transduction, AO—Antioxidant.

The results of pathway analysis of the DEGs identified according to the P-value cut-off (FDR) < 0.05 from the ShinyGO database are shown in Table 3. It was observed that the ‘biosynthesis of secondary metabolites’ pathway was common for all the three stages of cryopreservation stresses (T1, T2 and T3). Two pathways, ‘3-ketoacyl-CoA synthase 6-like’ and ‘plant-pathogen interaction’, were induced in T2 and T3. ‘MAPK signaling’, ‘EIN 3-like 1 protein’, ‘fatty acid elongation, ascorbate and aldarate metabolism’ and ‘biosynthesis of cofactors’ pathways were induced in T1 and T3. Some important pathways induced in T1-treated samples were ‘glycolysis/gluconeogenesis’, ‘tyrosine metabolism’, ‘alpha-linolenic acid metabolism’, ‘fatty acid degradation’, ‘pyruvate metabolism’, etc. (Figure 6). The comparative pathway analysis of the significant DEGs with control (T0) and T2 showed maximum number of genes involved in amino acid metabolism followed by pathways related to antioxidants (Figure 7A). However, the comparative pathway analysis between control (T0) with T3 showed a maximum number of genes enriched in KEGG pathways in secondary metabolites synthesis followed by fatty acid metabolism (Figure 7B).

## 3. Discussion

In general, the successful cryopreservation of shoot meristematic explants during vitrification-based techniques depends on a trade-off between dehydration stress (caused by high sucrose treatment), toxicity of CPAs and freezing injury of tissue (during LN treatment), and the cellular responses to these three stresses. While most of these cryotechniques overcome ice damage by glassy formation, other cryo-injuries due to oxidative stress, osmotic shock, disturbed ion homeostasis, altered physical and metabolic properties are known to occur, impacting the final outcome of regrowth of cryopreserved tissue [18]. To elucidate the molecular and cellular mechanisms during cryopreservation, the present study on the cryobiomics of banana meristem with very high regrowth after PVS2 treatment (95.0%) and LN (66.7%) [17] was selected using a RNA-seq approach. This is the first report in *Musa* where the transcriptome profiling of shoot meristem is carried out in different cryopreservation stages using RNA Seq analysis. 

Among the three stages of the DV cryoprotocol, pre-culturing with high sucrose (T1) led to the highest number of upregulated and downregulated DEGs (201) as compared to subsequent T2 (8) and T3 (13) treatments. Earlier work on the shoot tip cryopreservation of the model plant *Arabidopsis thaliana* using microarray analysis revealed that a total of 74 transcripts responded to PVS2 treatment, but only two transcripts were differentially expressed as a result of the subsequent LN exposure [15]. However, in the same plant, Ren et al. [19] using microarray analysis reported that 941 genes are differentially expressed by dehydration treatment (PVS2) and 3,970 DEGs are found after LN treatment followed by thawing and culture on regrowth medium. In *Arabidopsis*, functional transcripts in response to cryopreservation were predominantly related to gene ontologies for stress, defense, wounding, carbohydrate metabolism, lipid metabolism, osmoregulation, oxidation and temperature [15].

### 3.1. Common DEGs in All Phases of Cryopreservation, as Compared to Control Tissue

Among the 70 DEGs co-expressed in all the phases of treatment in cryopreservation, 36 were upregulated and 34 were downregulated (Supplementary Table S2). Interestingly, most of them are consistent in their fold increase among the treatments. Among the 36 common upregulated DEGs, the highest (>20-fold) increase occurred in transferase activity (glycan metabolism) and the GRAS domain-containing protein (regulation of transcription) in all three stages, while calcium ion binding proteins expressed a variation in fold increase among the three stages (Supplementary Table S2). 

UDP glycosyltransferases (UGTs) in plants have been ascribed to participate in the regulation of cellular homeostasis and detoxification of xenobiotics [19] as well as the biosynthesis and transport properties of triterpenoid [20]. In plants, triterpenoids have several roles including stress and defense responses, antioxidant activity and thermotolerance [7,21,22]. Hence, the upregulation of genes for transferase activity (glycan metabolism) with a 20-fold increase at the transcript level in the present study during all stages of cryopreservation reveals that SMs are produced in response to osmotic, desiccation and cold stresses for maintaining cellular homeostasis and combating reactive oxygen species (ROS) that are known to increase during the process of cryopreservation [7]. *Vitis amurensis VaPAT1* (Phytochrome A signal transduction 1), a member of the GRAS Transcription Factor (TF) family, has been shown to be involved in response to cold, drought and salinity tolerance by modulating the expression of stress-related genes such as *LOX3* (lipoxygenase 3), which is a gene encoding jasmonate biosynthesis [5,23]. During the cryopreservation of tissue in the present work, the upregulation of GRAS domain-containing protein could possibly be involved in enhancing the further downstream activation of stress response genes through its role of primarily regulation of transcription factors. Low-temperature treatment triggers Ca^2+^ transients through Ca^2+^ channels and/or Ca^2+^ pumps [24], and the response to stress perception is increased with the intracellular accumulation of free Ca^2+^, which serves as a second messenger to induce stress response gene expression [25]. In the current findings, the expression of calcium ion-binding proteins showed a two-fold increase in T2 (PVS2) and T3 (LN) treatments compared to high sucrose treatment (T1), which further regulates genes in response to CPAs and freeze tolerance.

Among the 34 common downregulated DEGs, oxidoreductase activity was higher fold underexpressed in all three stages, while GRAM domain-containing protein and acyl carrier protein showed a variation in expression among the three stages (Supplementary Table S2). Oxidoreductase activity, especially membrane enzyme glycerol-3 phosphate dehydrogenase, is involved in respiration and energy metabolism [26]. The >20-fold downregulation in all the treatment stages (T1, T2, T3) indicates the adjustment of energy reactions of the cells under anoxia condition due to osmotic, desiccation and freezing stress during cryopreservation. 

In the present work, gene encoding for the GRAM domain-containing protein (part of glucosyltransferases and other membrane-associated proteins) showed a 10-fold decrease in T1 and T3 but 5-fold decrease in T2, which indicates their differential regulation to different signals stimulation. The GRAM domain has been reported to be an intracellular protein binding or lipid binding signaling domain, which has an important function in membrane-associated processes [27]. Many GRAM domain family members are known for their divergent functional response to environmental (cold, drought and salinity) and hormonal signaling (ABA) [28,29]. Thus, the downregulation of the GRAM domain-containing protein during cryopreservation would likely be to avoid cell membrane damage during osmotic pressure and low temperature.

The acyl carrier protein in chloroplast plays a major role in the biosynthesis of fatty acids for membrane lipids [30]. The three-fold downregulation of this gene in PVS2 treatment (T2) compared to T1 and T3 indicates the cell’s reaction to its exposure to cryoprotectant (PVS2), which contains both penetrating (DMSO) and non-penetrating (ethylene glycol) chemicals that affect the change of water content in the cells. Lin et al. [31] have shown that that the remodeling of membrane lipids and attenuation of lipid degradation are critical for the successful use of CPAs (such as PVS2) in a cryoprotocol, wherein both the lipid content and membrane composition were not static during cryopreservation but remodeled at each step in the process to reflect the specific stress being imposed. Our work also supports this hypothesis.

### 3.2. Differently Expressed Genes during High-Sucrose Phase

Earlier reports on the cryopreservation of banana-proliferating meristems clearly indicated the crucial role of pre-culture on high-sucrose medium (0.4 and 0.5 M) to obtain significantly better survival (12–72%) after LN treatment [32,33,34]. Meristems pre-cultured on medium with normal sucrose (0.1 M) led to no survival after cryopreservation in all the seven banana accessions tested, indicating the very critical role of the sucrose pre-culture plant. Panis et al. [32,35] postulated that pre-culturing on high sucrose enhances the desiccation of cells, preventing intra- and extra-cellular ice crystal formation while also protecting the structural integrity of cell membranes by preventing membrane fusion, phase transition and phase separation. 

The role of sucrose in banana cryopreservation is re-affirmed in the present study, as transition from T0 to T1 produced multiple and the highest number of DEGs compared to the subsequent T2 and T3 treatments (Supplementary Table S3). The DEGs EIN 3-like 1 protein, GRAS domain-containing protein, very-long-chain 3-ketoacyl-CoA synthase and water channel activity were significantly upregulated. The EIN3 target genes encode TFs (several ERFs such as ERF1) which bind to dehydration-responsive elements and regulate response to abiotic stresses, including a range of ethylene responses such as CBF1/2/3, which is the regulator of cold stress responses [36,37,38]. The ten-fold increase in very-long-chain 3-ketoacyl-CoA synthase which regulates the fatty acid biosynthesis pathway may participate in enhancing the membrane integrity related to osmotic stress during high-sucrose pretreatment [39]. The activation of *EIN3* and members of the GRAS families TFs during high-sucrose pre-culture in the present work indicates their role in response to oxidative stress [25,40].

The gene responsible for DNA-binding protein (e.g., zinc finger domain-containing proteins) and GTP-binding protein were downregulated up to twenty-fold depicting the slowing down the molecular function (Supplementary Table S3). This could be possibly due to (i) the low availability of suitable substrate or requirements and (ii) the impaired function of osmotic/dehydration stress-related enzymes by high-sucrose treatment (T1) as osmotic or oxidative stress can damage DNA, proteins or macromolecules, and the cell membrane [41]. The membrane-related protein responsible for water channel activity has been found downregulating (11-fold) due to the non-availability of water molecules due to osmolyte accumulation. 

In GO functional classification (Figure 6A), upregulated DEGs from T0 to T1 were mostly associated with transmembrane transporter and antiporter activities especially in vacuoles and vacuole membranes to adjust the cellular homeostasis. Cell vacuoles serve as reservoirs in the release of protons and other metabolically important ions, e.g., Ca^2+^ into the cytosol for controlling pH and ionic homeostasis [42], and it seems that the vacuolar cation/proton exchanger confers calcium-proton antiporter activity in the balancing of cellular activities by adjusting the osmotic dehydration during high-sucrose pre-culturing. Furthermore, the present data also show an induction of alcohol dehydrogenase (ADH) and glutathione dehydrogenase (GSH-DH) activities to cope with formaldehyde and/or aldehyde-related biological processes, which are known to have roles in enhanced oxidative respiration (demanding energy) and sequestering reactive oxygen species (ROS), respectively [14].

The majority of downregulated DEGs were mostly associated with oxidoreductase and carbohydrate-binding activities and oxidation reduction, regulation of biological quality, and metabolic processes, as shown in Figure 6B. In response to osmotic stress, meristem cells divert substantial resources (e.g., carbon dioxide, nutrients and energy) to prevent or repair damage caused by stress to maintain cellular homeostasis, and that may decrease cellular metabolic processes and enzymes activities. 

Among the identified KEGG pathways, the upregulation and downregulation of DEGs are involved in primary and secondary metabolic pathways, the glycolysis/glucogenesis pathway, MAPK signaling pathway and plant hormone signal transduction pathway, etc. (Table 3). Our findings are similar to those of Zorrilla-Fontanesi et al. [43], who studied transcriptomic changes using mRNA-seq for analysis in banana root tips and reported that mild osmotic stress leads to a lower energy level, which induces a metabolic shift toward higher oxidative respiration, alternative respiration and fermentation. Since white proliferating shoot tip meristems were used in our study, their energy demand may shift from primarily aerobic respiration (which depends on glycolysis and mitochondrial respiration) to anaerobic respiration via fermentation through the activation of fermentative enzymes such as ADHs (ADH1 and ADH3) (Figure 7A; Supplementary Table S3). The induction of the fermentative enzymes (ADHs) is not exclusively dependent on the oxygen concentration, but it is also linked to changes in the energy status (ratio of ATP to ADP). Consequently, sensing the energy status would be an important component for optimizing plant metabolism, as the induction of ADHs is reported in increasing the transcript levels of multiple stress-related genes and the accumulation of soluble sugars [44]. Shinozaki et al. [45] have shown that ROS can be detected through the ROS receptors, Ca^2+^signalling, protein kinase and MAPK signaling cascades, hormone signaling, redox-sensitive TFs and phosphatases. The upregulation of oxidative stress response genes (e.g., EIN3 and GRAS) in banana meristems during high-sucrose pre-culture indicates adaptation and survival mechanisms by the tissue against the ROS generated during this phase. 

### 3.3. Differently Expressed Genes during PVS2 Phase

During the second treatment (T2) where the dehydrated cells were prepared for subsequent ultra-low temperature treatment using vitrification solution (PVS2), there were very few gene transcripts which were significantly upregulated (3) and downregulated (5) compared to T1 (Supplementary Table S3). 

Putative polyol transporter 1 is a plasma membrane sugar-proton symporter that exhibited a high fold increase in this treatment. Yamada and Osakabe [46] have suggested sugar compartmentation, which is mediated by sugar transporters, as an adaptation strategy against abiotic stresses (e.g., cold or drought) in plants. Thus, fluctuated sugar flow may have a special function in the optimization of cellular metabolites adjustment in response to PVS2, which also has high sucrose (0.4 M). Zinc finger A20 and AN1 domain (stress-associated protein (SAP) gene family) and C3H1-type domain-containing protein (type of zinc finger) have been analyzed in response to abiotic stresses (cold, drought, heat, salt, wounding, oxidative, ABA and submergence) in many plants [47,48,49,50]. The overexpression of these two genes in response to PVS2 treatment indicates their role in tolerance to severe desiccation, oxidative and chemical toxicity of PVS2 and may be involved in recovery from stress-induced injuries. Significantly downregulated DEGs include membrane-localized proteins (such as transmembrane 9 superfamily member and very-long-chain 3-ketoacyl-CoA synthase) and indicate that PVS2 treatment potentially causes membrane integrity loss. 

In functional classification, binding activities (metal ion, cation and DNA binding) are enriched, while other DNA-binding TFs and membrane-related molecular function were under-represented (Figure 6C). In KEGG pathway annotation (Table 3), membrane-related 3-ketoacyl-CoA synthase 6-like and the biosynthesis of secondary metabolites pathways were regulated (Figure 7). Our data support other findings such as the JA-mediated formation of secondary metabolites in plant stress responses [51], carotenoid biosynthesis functioning of pigmentation and antioxidant activity in the cryopreservation of *Arabidopsis* [7], and in stabilizing the membrane by adjusting secondary metabolites formation in thermotolerance in banana [21]. 

### 3.4. Differently Expressed Genes during LN Phase

The exposure to ultra-low temperature was the last step of cryopreservation where the cells with vitrification solution were subjected to LN (−196 °C). In Supplementary Table S3, upregulated and downregulated DEGs in PVS2 + LN (T3) are shown. Physical and chemical reactions and time-related changes are known to be almost arrested at ultra-low temperatures [10,52]. High-fold expressed DEGs were obtained for (i) nuclear transcription activator (factor Y subunit C-6-like protein) to regulate gene expression; (ii) very-long-chain 3-oxoacyl-CoA synthase for fatty acid biosynthesis to control membrane stability and (iii) nucleolar GTP-binding protein 1-like. Studies in *Arabidopsis* by Lee et al. [53] revealed that the NOG1-2 (nucleolar GTP-binding) protein regulates signaling in response to biotic and abiotic stimulus by interacting with jasmonic acid (JA) and abscisic acid (ABA). 

In contrast to tissues of T1 (sucrose pre-culture) where ~27 fold increase occurred, post-LN tissue (T3) gave about a 40-fold downregulation of the EIN 3-like 1 protein in the ethylene-activated signaling pathway and also a higher fold downregulation of auxin transport protein BIG, translation terminator-eRF1_3 domain-containing protein, carbohydrate binding-L-type lectin-domain containing receptor kinase VIII.1-like and stress responsive protein-C3H1-type domain-containing protein (type of zinc finger) to arrest most metabolic activities. Functional annotation shows that enzyme activities involved in the integral components of the membranes were enriched such as very-long-chain 3-ketoacyl-CoA synthase activity and 3-oxo-related-CoA synthase activities especially for fatty acid biosynthesis (Supplementary Table S3). 

In pathway analysis, 11 pathways were identified (Table 3). Although only the biosynthesis of the secondary metabolites pathway was regulated in all treatments (T1, T2 and T3), five pathways of EIN 3-like 1 protein, biosynthesis of cofactors, MAPK signaling pathway, ascorbate and aldarate metabolism and fatty acid elongation were common in T1 and T3. Fatty acid synthesis was involved in the structural modification of the cell membrane and organelle membrane, which is considered to be the primary site of freezing injury. The general desaturation of these lipids ensures that the membranes will remain as fluid as possible during freezing and thawing processes. Plants that have a better ability to cold acclimate also respond better at the molecular level to express and activate the enzymes required for the desaturation or hydrolysis of fatty acids. If this adjustment does not take place as the temperature is decreased, significant structural damage in cell membranes will occur.

## 4. Conclusions

This is a first report of transcriptome profiling of shoot meristem tissues in banana (*Musa* sp. AAA cv Borjahaji) through RNA seq, which was carried out in four different stages of droplet-vitrification cryopreservation protocol. The study provides the contribution of different metabolic genes in successive stages of cryopreservation of meristem cells. The results have shown that the number of DEGs related to membrane lipid remodeling and fatty acid unsaturation (very-long-chain 3-oxoacyl-CoA synthase), as well as stress responsive transcription activators (GRAS, AP2/ERFs, NAC, bZIP and HSFs families), were significantly expressed differentially in sequential stages of cryopreservation. Furthermore, the enhanced expression of energy-conserving glycolysis genes (ADHs) was recorded. Future comparative studies of different genotypes with varying response will provide gene targets or substrate requirement for greater success after cryopreservation, besides gene function validation using qRT-PCR. Such studies would be helpful in developing generalized protocol for *Musa* germplasm. 

## 5. Material and Methods

### 5.1. Plant Material

An Indian accession of triploid cultivated banana ‘Borjahaji’ (IC250462) with the AAA genome (Cavendish subgroup) was used in the present study. In vitro cultures were sourced from the In vitro Genebank of ICAR-National Bureau of Plant Genetic Resources, New Delhi, India, and the material was originally collected from the northeastern region of the country (http://pgrinformatics.nbpgr.ernet.in/akmu/Default.aspx) accessed on 20 October 2019. 

### 5.2. Cryopreservation

**Figure 8:**
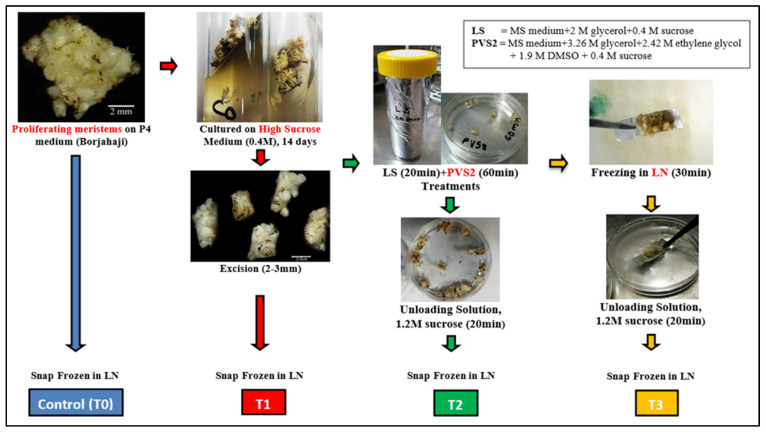
Preparation of samples for transcriptomes study in selected *Musa* (AAA) accession: Borjahaji in different stages of cryopreservation (Droplet vitrification): T0 (Control), T1 (High sucrose treatment), T2 (PVS2 treatment), T3 (LN treatment).

To study the differential gene expression, a droplet-vitrification cryotechnique was applied on proliferating meristems of the selected banana accession based on the protocol of Htwe et al. [17]. White cauliflower-like proliferating meristems raised on the P4 medium [54] salts + 100 μM 6-benzylaminopurine (BAP) + 1 μM Indole-3-acetic acid (IAA) + 1 µM ascorbic acid + 3% sucrose (0.09 M) + 0.25% Phytagel™] were subjected to dehydration prior to cryopreservation, and tissues at four sequential steps were used for RNA seq analysis (Table 4). A total of 8 samples including duplicates from T0, T1, T2, and T3 (approximately 2 g each) were flash-frozen in LN immediately to avoid RNA degradation and then kept at −80 °C Deep Freezer until the subsequent RNA extraction (Figure 8).

For regrowth after cryopreservation, explants were placed on two sterile filter papers (Whatman No. 1) on top of semi-solid hormone-free MS medium (recovery medium) containing 0.3 M sucrose in a Petri dish and incubated in the dark for 48 h. Then, meristems were transferred to the semi-solid regeneration (C3) medium (MS medium + 2.22 μM BA + 0.09 M sucrose + 0.25% phytagel) without filter papers and held in the dark for 14 d. Thereafter, meristems were transferred to the P6 regeneration medium (MS medium + 0.09 M sucrose +1 μM BAP + 0.25% phytagel) and kept in 16 h light/8 h dark (40 μMm^−2^ s^−1^) at 25 ± 2 °C [17]. Shoot regeneration was recorded after six weeks as the percentage of explants surviving the cryopreservation treatment.

### 5.3. Transcriptome Analysis

#### 5.3.1. RNA Extraction

The experiment for transcriptome analysis was set up as a balanced block design CRD with two biological replications for four treatments including proliferating meristems growing on P4 medium as a control (Table 4). About 80–100 clusters of meristems (2–3 mm) weighing approximately 2 g were used for total RNA isolation. Total RNA was isolated using conventional TRIzol^®^ method with minor modifications [55]. The quality and quantity of isolated RNA were checked by electrophoresis (1% denaturing RNA agarose gel) and nanophotometer (NanoDrop^TM^, ThermoFischer Scientific, Waltham, MA, USA), respectively. The quality of RNA was checked through Agilent 2100 Bioanlyser, Germany before library preparation.

#### 5.3.2. cDNA Library Preparation and Sequencing

Ribonucleic acid paired-end sequencing (RNA-Seq) libraries were prepared from the quality check (QC passed) RNA samples using a TruSeq^TM^ stranded mRNA sample prep kit (Illumina Inc., San Diego, CA, USA). The mRNAs were enriched from the total RNA using Poly-T attached magnetic beads, which was followed by enzymatic fragmentation, 1st strand cDNA conversion using SuperScript II and Act-D mix to facilitate RNA-dependent synthesis. The 1st strand cDNA was then synthesized to second strand using a second strand mix. Thereafter, dscDNA (double-stranded cDNA) was purified using AMPure XP beads followed by A-tailing, adapter ligation and followed by enrichment by PCR cycles. The PCR-enriched libraries were purified using AMPureXP beads and analyzed on a 4200 Tape Station system (Agilent Technologies, Santa Clara, CA, USA).

A total of eight cDNA libraries were prepared for the four different sample stages (T0, T1, T2, T3) in biological replicates. Paired-end sequencing was performed by an Illumina NovaSeq6000 sequencer (Illumina Inc., San Diego, CA, USA.) to obtain raw reads. As raw reads, the data gave high-quality results, and adaptor sequences were trimmed by Cutadapt [56]. All the downstream analyses were performed only with high-quality cleaned and trimmed data. Clean reads were mapped to the *Musa acuminata* DH Pahang reference genome (https://banana-genome-hub.southgreen.fr/content/m-acuminata-dh-pahang-version-1 accessed on 5 March 2021) using the Hisat2 program (Hierarchical Indexing for Spliced Alignment of Transcripts) [57,58]. Before mapping, the indexed reference genome was prepared by Hisat2-build. To determine gene expression level, the quantification of fragments per kilobase of transcript per million reads (FPKM) was performed using the StringTie tool [58,59]. FPKM values of all genes from different stages and their biological replicates were used to build a PCA plot using iDEP.96 Integrated Differential Expression and Pathway Analysis (http://bioinformatics.sdstate.edu/idep/ accessed on 5 March 2021).

Analysis for differentially expressed genes (DEGs) of all treatments (T1, T2, T3) as compared to control (T0) and among the different treatments with two biological replicates of each set was performed using DESeq2 [60]. The R package transcripts having a P value of 0.05 and a log-fold change threshold equal to 1 were considered as DEGs. Transcripts having Padj < 0.05 were considered significant DEGs. These were further classified on the basis of logFC > 2.0 taken as upregulated and logFC < −2.0 considered as downregulated. The GO (Gene Ontology) annotation for all significant DEGs was gathered from the UniProt database, and GO enrichment was carried out by the ShinyGo Database [61]. Enrichment analysis of all significant data was performed with a P value cut-off (FDR) of 0.05, and the top 100 pathways were taken into account for functional categories such as BP (Biological Process), CC (Cellular Component) and MF (Molecular Function). For pathway enrichment, the KEGG (Kyoto Encyclopedia of Genes and Genomics) database was used [62].

## Figures and Tables

**Table 1:** Statistics of the cleaned transcript sequence data and its alignment rate after mapping with reference genome, obtained in the tissue from four stages of cryopreservation (in two biological replicates).

Treatment Stage and No. for RNA Seq Analysis	Biological Replicate	No. of Clean Reads	GC%	Alignment Rate with Reference Genome (%)
T0—Control tissue	1	32,646,264	48	84.31
	2	42,178,012	50	89.48
T1—High-sucrose (0.4 M) treated tissue	1	26,508,752	48	72.06
	2	45,680,016	51	91.13
T2—PVS2-treated control tissue	1	7,506,720	47	85.55
	2	42,852,004	49	87.62
T3—LN-treated tissue	1	44,515,874	53	93.12
	2	44,965,434	51	92.40

**Table 2:** Statistics of significant Differentially Expressed Genes (DEGs).

	Significant DEGs (Sig Padj < 0.05)	Upregulated (log FC > 2.0)	Downregulated (log FC < −2.0)
T1 (0.4 M high sucrose-treated tissue) vs. T0 (control)	201	79	122
T2 (PVS2-treated tissue) vs. T0	213	107	106
T3 (LN-treated tissue) vs. T0	132	58	74
T2 vs. T1	8	3	5
T3 vs. T2	13	4	9
T3 vs. T1	17	7	10

**Table 3:** Annotation of significant DEGs associated with KEGG pathways of subsequent stages of cryopreservation.

Stage I (T1 vs. T0)	Stage II (T2 vs. T1)	Stage III (T3 vs. T2)
Biosynthesis of secondary metabolites	Biosynthesis of secondary metabolites	Biosynthesis of secondary metabolites
Alpha-linolenic acid metabolism	3-ketoacyl-CoA synthase 6-like	3-ketoacyl-CoA synthase 6-like
Glycolysis/Gluconeogenesis	Plant–pathogen interaction	Plant-pathogen interaction
Pyruvate metabolism	Fatty acid elongation	UDP-glucose 6-dehydrogenase 4
Biosynthesis of cofactors		Biosynthesis of cofactors
Ascorbate and aldarate metabolism		Ascorbate and aldarate metabolism
Fatty acid elongation		Fatty acid elongation
MAPK signaling pathway		MAPK signaling pathway
ETHYLENE INSENSITIVE 3-like 1 protein		ETHYLENE INSENSITIVE 3-like 1 protein
Plant hormone signal transduction		Ribosome biogenesis in eukaryotes
Amino sugar and nucleotide sugar metabolism		Nucleolar GTP-binding protein 1-like
Starch and sucrose metabolism		
Linoleic acid metabolism		
Galactose metabolism		
Fatty acid degradation		
Glycerophospholipid metabolism		
Biosynthesis of amino acids		
Pentose phosphate pathway		
Glutathione metabolism		
Tyrosine metabolism		
Protein processing in endoplasmic reticulum		
Citrate cycle (TCA cycle)		
Diterpenoid biosynthesis		
Phenylpropanoid biosynthesis		
Cysteine and methionine metabolism		
Glycerolipid metabolism		
mRNA surveillance pathway		
Inositol phosphate metabolism		
Other glycan degradation		
Glyoxylate and dicarboxylate metabolism		
Carotenoid biosynthesis		

**Table 4:** Various stages of droplet-vitrification cryotechnique at which meristematic tissue samples were used for RNA seq analysis.

	Steps for Cryopreservation	Treatment Stage and no. for RNA Seq Analysis
(i)	Proliferating meristems (in clumps on corm tissue of 6–10 mm diameter) growing on P4 medium (4 weeks old).	T0—Control tissue
(ii)	Above tissues subjected to pre-culture desiccation by transferring on C0 medium (MS salts + 10 μM BAP + 1 μM IAA + 1 µM ascorbic acid + 13.9% sucrose (0.4 M) + 0.25% Phytagel™) for 2 weeks and incubated at 25 ± 2 °C in the dark.	T1—High-sucrose-treated tissue
(iii)	Small meristematic clumps containing at least 3–5 apical domes (2.3 mm diameter) excised from pre-cultured clusters and placed in a plastic container containing loading solution (LS): 2M glycerol + 0.4 M sucrose in MS medium with pH 5.8 for 20 min at 25°C. After treating with LS solution, explants treated with ice-cold, filter-sterilized plant vitrification solution 2 PVS2: 30% (3.26 M) glycerol + 15% (2.42 M) ethylene glycol + 15% (1.9 M) dimethyl sulfoxide (DMSO) + 0.4 M sucrose in MS medium (pH 5.8) at 0 °C for 60 min. Thereafter, PVS2-treated explants were rinsed in 10 mL of unloading solution (RS) containing MS medium supplemented with 1.2 M sucrose at pH 5.8 at room temperature (RT) for 20 min.	T2—LS and PVS2-treated tissue
(iv)	Small meristematic clumps from C0 medium were treated with LS solution and PVS2 solution in similar procedures to step (iii) above. Five min before the completion of the incubation period (60 min) in PVS2, meristematic clumps were transferred to a drop of fresh, chilled PVS2 solution placed on a strip of pre-autoclaved aluminum foil (20 × 5 mm) and plunged directly into liquid nitrogen (LN) for at least 30 min. After exposure to LN, the explants with aluminum foil strips were taken out and rapidly rinsed in 10 mL of RS at RT for 20 min.	T3—LN-treated tissue

## Data Availability

RNA-seq data have been deposited to NCBI database, with the accession number of PRJNA801423.

## Supplementary Materials

The following supporting information can be downloaded at: https://www.mdpi.com/article/10.3390/plants12051165/s1, Table S1: Details of differentially expressed genes in all three stages of cryopreservation protocol compared to control; Table S2: Details of common of differentially expressed genes in all three stages of cryopreservation protocol; Table S3: Details of differentially expressed genes in sequential stages of cryopreservation protocol.

## References

[1] Singh S., Malhotra E.V., Meena D.P.S., Tyagi R.K., Rajasekharan P., Rao V. (2019). In Vitro Conservation and Cryopreservation of Clonally Propagated Horticultural Species. Conservation and Utilization of Horticultural Genetic Resources.

[2] Martinez-Montero M.E., Harding K., Barh D., Khan M., Davies E. (2015). Cryobionomics: Evaluating the Concept in Plant Cryopreservation. PlantOmics: The Omics of Plant Science.

[3] Wyse S.V., Dickie J.B., Willis K.J. (2018). Seed banking not an option for many threatened plants. Nat. Plants.

[4] Niino T., Arizaga M.V. (2015). Cryopreservation for preservation of potato genetic resources. Breed. Sci..

[5] Wang M.R., Lambardi M., Engelmann F., Pathirana R., Panis B., Volk G.M. (2021). Advances in cryopreservation of in vitro-derived propagules: Technologies and explant sources. Plant Cell Tissue Organ Cult..

[6] Bettoni J.C., Bonnart R., Volk G.M. (2021). Challenges in implementing plant shoot tip cryopreservation technologies. Plant Cell Tissue Organ Cult..

[7] Ren L., Zhang D., Chen G., Reed B.M., Shen X., Chen H. (2015). Transcriptomic profiling revealed the regulatory mechanism of *Arabidopsis* seedlings response to oxidative stress from cryopreservation. Plant Cell Rep..

[8] Whelehan L.M., Funnekotter B., Bunn E., Mancera R.L. (2022). Review: The case for studying mitochondrial function during plant cryopreservation. Plant Sci..

[9] Panis B., Van den houwe I., Piette B., Swennen R. (2007). Cryopreservation of the banana germplasm collection at the International Transit Centre—Bioversity International. Adv. Hort. Sci..

[10] Agrawal A., Tyagi R.K. (2014). In vitro conservation and cryopreservation of genetic resources of *Musa* spp. A review of the recent developments with special reference to India. Int. J. Innov. Hortic..

[11] Singh S., Agrawal A., Kumar R., Thangjam R., Joseph John K. (2021). Seed storage behavior of *Musa balbisiana* Colla, a wild progenitor of bananas and plantains—Implications for ex situ germplasm conservation. Sci. Hortic..

[12] Panis B., Piette B., Swennen R. (2005). Droplet vitrification of apical meristems: A cryopreservation protocol applicable to all Musaceae. Plant Sci..

[13] Uma S., Saraswathi M.S., Backiyarani S., Durai P., Agrawal A., Uma S., Vaganan M.M., Agrawal A. (2020). Unravelling the potential of *Musa* genetic resources for benefit of global banana community. Banana & Plantains: Leading-Edge Research and Developments. Volume 1: Germplasm Diversity, and Breeding.

[14] Volk G.M., Henk A., Basu C. (2011). Gene expression in response to cryoprotectant and liquid nitrogen exposure in *Arabidopsis* shoot tips. Acta Hortic..

[15] Gross B.L., Henk A.D., Bonnart R., Volk G.M. (2017). Changes in transcript expression patterns as a result of cryoprotectant treatment and liquid nitrogen exposure in *Arabidopsis* shoot tips. Plant Cell Rep..

[16] Stock J., Bräutigam A., Melzer M., Bienert G.P., Bunk B., Nagel M., Overmann J., Keller E.R.J., Mock H.P. (2020). The transcription factor WRKY22 is required during cryo-stress acclimation in *Arabidopsis* shoot tips. J. Exp. Bot..

[17] Htwe C.S.S., Singh H., Agrawal A. (2021). Comparison of three cryotechniques for conservation of banana (*Musa* AAA, Cavendish Subgroup) genotypes. Indian J. Plant Genet. Resour..

[18] Chen G., Zhang D., Pan J., Yue J., Shen X. (2021). Cathepsin B-like cysteine protease ApCathB negatively regulates cryo-injury tolerance in transgenic *Arabidopsis* and *Agapanthus praecox*. Plant Sci..

[19] Srivastava P., Garg A., Misra R.C., Chanotiya C.S., Ghosh S. (2021). UGT86C11 is a novel plant UDP-glycosyltransferase involved in labdane diterpene biosynthesis. J. Biol. Chem..

[20] Khorolragchaa A., Kim Y.J., Rahimi S., Sukweenadhi J., Jang M.G., Yang D.C. (2014). Grouping and characterization of putative glycosyltransferase genes from *Panax ginseng* Meyer. Gene.

[21] Vidya S.M., Kumar H.S.V., Bhatt R.M., Laxman R.H., Ravishankar K.V. (2018). Transcriptional profiling and genes involved in acquired thermotolerance in Banana: A non-model crop. Sci. Rep..

[22] Yu J., Hu F., Dossa K., Wang Z., Ke T. (2017). Genome-wide analysis of UDP-glycosyltransferase super family in *Brassica rapa* and *Brassica oleracea* reveals its evolutionary history and functional characterization. BMC Genom..

[23] Xin H., Zhu W., Wang L., Xiang Y., Fang L., Li J. (2013). Genome wide transcriptional profile analysis of *Vitis amurensis* and *Vitis vinifera* in response to cold stress. PLoS ONE.

[24] Mori K., Renhu N., Naito M., Nakamura A., Shiba H., Yamamoto T. (2018). Ca (2+)-permeable mechanosensitive channels MCA1 and MCA2 mediate cold-induced cytosolic Ca(2+) increase and cold tolerance in *Arabidopsis*. Sci. Rep..

[25] Lohani N., Jain D., Singh M.B., Bhalla P.L. (2020). Engineering multiple abiotic stress tolerance in canola, *Brassica napus*. Front. Plant Sci..

[26] Shen W., Wei Y., Dauk M., Zheng Z., Zou J. (2003). Identification of a mitochondrial glycerol-3-phosphate dehydrogenase from *Arabidopsis thaliana*: Evidence for a mitochondrial glycerol-3-phosphate shuttle in plants. FEBS Lett..

[27] Lorrain S., Lin B., Auriac M.C., Kroj T., Saindrenan P., Nicole M. (2004). Vascular associated death1, a novel GRAM domain-containing protein, is a regulator of cell death and defense responses in vascular tissues. Plant Cell.

[28] Jiang S.Y., Ramamoorthy R., Ramachandran S. (2008). Comparative transcriptional profiling and evolutionary analysis of the GRAM domain family in eukaryotes. Dev. Biol..

[29] Tiwari S., Shweta S., Prasad M., Lata C. (2020). Genome-wide investigation of GRAM-domain containing genes in rice reveals their role in plant-rhizobacteria interactions and abiotic stress responses. Int. J. Biol. Macromol..

[30] Branen J.K., Shintani D.K., Engeseth N.J. (2003). Expression of antisense acyl carrier protein-4 reduces lipid content in Arabidopsis leaf tissue. Plant Physiol..

[31] Lin L., Ma J., Ai Q., Pritchard H.W., Li W., Chen H. (2021). Lipid remodeling confers osmotic stress tolerance to embryogenic cells during cryopreservation. Int. J. Mol. Sci..

[32] Panis B., Totté N., Van Nimmen K., Withers L.A., Swennen R. (1996). Cryopreservation of banana (*Musa* spp.) meristem cultures after preculture on sucrose. Plant Sci..

[33] Carpentier S.C., Witters E., Laukens K., Van Onckelen H., Swennen R., Panis B. (2007). Banana (*Musa* spp.) as a model to study the meristem proteome: Acclimation to osmotic stress. Proteomics.

[34] Carpentier S.C., Vertommen A., Swennen R., Witters E., Fortes C., Souza J.M.T., Panis B. (2010). Sugar-mediated acclimation: The importance of sucrose metabolism in meristems. J. Proteome Res..

[35] Panis B., Strosse H., Van Den Hende S., Swennen R. (2002). Sucrose preculture to simplify cryopreservation of banana meristem cultures. Cryo Lett..

[36] Leyser O., Day S., Buchanan B., Gruissem W., Jones R.L. (2015). Signal transduction. Biochemistry & Molecular Biology of Plants.

[37] Müller M., Munné-Bosch S. (2015). Ethylene response factors: A key regulatory hub in hormone and stress signaling. Plant Physiol..

[38] Dolgikh V.A., Pukhovaya E.M., Zemlyanskaya E.V. (2019). Shaping ethylene response: The role of EIN3/EIL1 transcription factors. Front. Plant Sci..

[39] Raffaele S., Leger A., Roby D. (2009). Very long chain fatty acid and lipid signaling in the response of plants to pathogens. Plant Signal. Behav..

[40] Asensi-Fabado M.A., Cela J., Müller M., Arrom L., Chang C., Munné-Bosch S. (2012). Enhanced oxidative stress in the ethylene-insensitive (ein3-1) mutant of *Arabidopsis thaliana* exposed to salt stress. J. Plant Physiol..

[41] Best B.P. (2015). Cryoprotectant toxicity: Facts, issues, and questions. Rejuvenation Res..

[42] Staehelin L.A., Guchanan B.B., Gruissem W., Jones R.L. (2015). Membrane structure and membranous organelles. Biochemistry & Molecular Biology of Plants.

[43] Zorrilla-Fontanesi Y., Rouard M., Cenci A., Kissel E., Do H., Dubois E. (2016). Differential root transcriptomics in a polyploid non-model crop: The importance of respiration during osmotic stress. Sci. Rep..

[44] Shi H., Liu W., Yao Y., Wei Y., Chan Z. (2017). Alcohol dehydrogenase 1 (ADH1) confers both abiotic and biotic stress resistance in *Arabidopsis*. Plant Sci. Int. J. Exp. Plant Biol..

[45] Shinozaki K., Uemura M., Bailey-Serres J., Bray E.A., Weretilnyk E., Buchanan B.B., Gruissem W., Jones R.L. (2015). Responses to abiotic stress. Biochemistry& Molecular Biology of Plants.

[46] Yamada K., Osakabe Y. (2018). Sugar compartmentation as an environmental stress adaptation strategy in plants. Semin. Cell Dev. Biol..

[47] Gao W., Long L., Tian X., Jin J., Liu H., Zhang H., Xu F., Song C. (2016). Genome-wide identification and expression analysis of stress-associated proteins (SAPs) containing A20/AN1 zinc finger in cotton. Mol. Genet. Genom..

[48] Li W., Wang Y., Li R., Chang X., Yuan X., Jing R. (2021). Cloning and characterization of TaSAP7-A, a member of the stress-associated protein family in common wheat. Front. Plant Sci..

[49] Solanke A.U., Sharma M.K., Tyagi A.K., Sharma A.K. (2009). Characterization and phylogenetic analysis of environmental stress-responsive SAP gene family encoding A20/AN1 zinc finger proteins in tomato. Mol. Genet. Genom..

[50] Han G., Qiao Z., Li Y., Wang C., Wang B. (2021). The roles of CCCH zinc-finger proteins in plant abiotic stress tolerance. Int. J. Mol. Sci..

[51] Wasternack C. (2014). Action of jasmonates in plant stress responses and development—Applied aspects. Biotechnol. Adv..

[52] Panis B., Nagel M., Van den Houwe I. (2020). Challenges and prospects for the conservation of crop genetic resources in field genebanks, in in vitro collections and/or in liquid nitrogen. Plants.

[53] Lee S., Rojas C.M., Oh S., Kang M., Choudhury S.R., Lee H.-K. (2018). Nucleolar GTP-Binding Protein 1-2 (NOG1-2) interacts with Jasmonate-ZIM Domain Protein 9 (JAZ9) to regulate stomatal aperture during plant immunity. Int. J. Mol. Sci..

[54] Murashige T., Skoog F. (1962). A revised medium for rapid growth and bio assays with tobacco tissue cultures. Physiol. Plant..

[55] Rio D.C., Ares M.J., Hannon G.J., Nilsen T.W. (2010). Purification of RNA using TRIzol (TRI reagent). Cold Spring Harb. Protoc..

[56] Martin M. (2011). Cutadapt removes adapter sequences from high-throughput sequencing reads. EMBnet J..

[57] Kim D., Langmead B., Salzberg S.L. (2015). HISAT: A fast spliced aligner with low memory requirements. Nat. Methods.

[58] Pertea M., Kim D., Pertea G.M., Leek J.T., Salzberg S.L. (2016). Transcript-level expression analysis of RNA-seq experiments with HISAT, StringTie and Ballgown. Nat. Protoc..

[59] Pertea M., Pertea G.M., Antonescu C.M., Chang T.C., Mendell J.T., Salzberg S.L. (2015). StringTie enables improved reconstruction of a transcriptome from RNA-seq reads. Nat. Biotechnol..

[60] Love M.I., Huber W., Anders S. (2014). Moderated estimation of fold change and dispersion for RNA-seq data with DESeq2. Genome Biol..

[61] Ge S.X., Jung D., Yao R. (2020). ShinyGO: A graphical gene-set enrichment tool for animals and plants. Bioinformatics.

[62] Kanehisa M., Goto S. (2000). KEGG: Kyoto encyclopedia of genes and genomes. Nucleic Acids Res..

